# Lopinavir, an HIV-1 peptidase inhibitor, induces alteration on the lipid metabolism of *Leishmania amazonensis* promastigotes

**DOI:** 10.1017/S0031182018000823

**Published:** 2018-05-28

**Authors:** Karina M. Rebello, Valter V. Andrade-Neto, Aline A. Zuma, Maria Cristina M. Motta, Claudia Regina B. Gomes, Marcus Vinícius N. de Souza, Geórgia C. Atella, Marta H. Branquinha, André L. S. Santos, Eduardo Caio Torres-Santos, Claudia M. d'Avila-Levy

**Affiliations:** 1 Laboratório de Estudos Integrados em Protozoologia, Instituto Oswaldo Cruz, Fundação Oswaldo Cruz (FIOCRUZ), Rio de Janeiro, Brazil; 2 Laboratório de Bioquímica de Tripanosomatídeos, Instituto Oswaldo Cruz, FIOCRUZ, Rio de Janeiro, Brazil; 3 Laboratório de Ultraestrutura Celular Hertha Meyer, Instituto de Biofísica Carlos Chagas Filho, Universidade Federal do Rio de Janeiro (UFRJ), Rio de Janeiro, Brazil; 4 Laboratório de Síntese de Fármacos, Farmanguinhos, FIOCRUZ, Rio de Janeiro, Brazil; 5 Instituto de Bioquímica Médica, UFRJ, Rio de Janeiro, Brazil; 6 Laboratório de Investigação de Peptidases, Instituto de Microbiologia Paulo de Góes, UFRJ, Rio de Janeiro, Brazil

**Keywords:** Aspartyl, chemotherapy, co-infection, leishmaniasis, trypanosomatids

## Abstract

The anti-leishmania effects of HIV peptidase inhibitors (PIs) have been widely reported; however, the biochemical target and mode of action are still a matter of controversy in *Leishmania* parasites. Considering the possibility that HIV-PIs induce lipid accumulation in *Leishmania amazonensis*, we analysed the effects of lopinavir on the lipid metabolism of *L. amazonensis* promastigotes. To this end, parasites were treated with lopinavir at different concentrations and analysed by fluorescence microscopy and spectrofluorimetry, using a fluorescent lipophilic marker. Then, the cellular ultrastructure of treated and control parasites was analysed by transmission electron microscopy (TEM), and the lipid composition was investigated by thin-layer chromatography (TLC). Finally, the sterol content was assayed by gas chromatography–mass spectrometry (GC/MS). TEM analysis revealed an increased number of lipid inclusions in lopinavir-treated cells, which was accompanied by an increase in the lipophilic content, in a dose-dependent manner. TLC and GC–MS analysis revealed a marked increase of cholesterol-esters and cholesterol. In conclusion, lopinavir-induced lipid accumulation and affected lipid composition in *L. amazonensis* in a concentration–response manner. These data contribute to a better understanding of the possible mechanisms of action of this HIV-PI in *L. amazonensis* promastigotes. The concerted action of lopinavir on this and other cellular processes, such as the direct inhibition of an aspartyl peptidase, may be responsible for the arrested development of the parasite.

## Introduction

Leishmaniasis is a tropical disease caused by an intracellular parasite of the genus *Leishmania*, which is transmitted by phlebotomine sand flies. Leishmaniasis is characterized by wide clinical presentations, ranging from cutaneous ulcers to deadly visceral lesions. An estimated 900 000–1.3 million new cases and 20 000–30 000 deaths occur annually (Alvar *et al.*, 2012). Concurrently, human immunodeficiency virus (HIV) infection is a major global public health problem and the prevalence is increasing worldwide. This scenario is worsened by an overlap between HIV and several infectious diseases, including leishmaniasis (Alvar *et al.*, 2008). As a result, the number of reported coinfection cases has been increasing globally (Lindoso *et al.*, 2016), and these patients have a higher risk of treatment failure, a higher risk of relapse and higher rates of mortality (Alvar *et al.*, 2008).

The current therapeutic approach to leishmaniasis is still based on a handful of drugs developed in the beginning and middle of the last century and that present several drawbacks, such as high costs, mode of administration and resistance emergence. The available compounds are pentavalent antimonials, amphotericin B, pentamidine, miltefosine and paromomycin. Although it is clear that the aetiological agent plays a crucial role in the clinical disease patterns, the choice of treatment is mainly based on drug availability in each country or region and patient care availability, since several drugs require hospitalization due to intravenous administration route and the severe side-effects. Also, there are no easily accessible methods for species-specific diagnosis in endemic areas, which reduces the physician possibility to choose the most suitable chemotherapeutic approach. Although miltefosine, which has been recently approved by US Food and Drug Administration (FDA), has the tremendous advantage of being orally administered, its teratogenic potential and the long half-life that can lead to the selection of drug-resistant lines still raises doubts on its suitability. The overlap between HIV and leishmaniasis poses additional problems to patient treatment, mainly due to the lack of information on chemotherapeutic combination (reviewed by Uliana *et al.*, 2017). It is clear that the main problems related to leishmaniasis treatment are mainly economical (Ennes-Vidal *et al.*, 2017; Uliana *et al.*, 2017; Alvar and Arana, 2018).

The antiretroviral treatment has a tremendous impact in the development of acquired immunodeficiency syndrome; it delays relapses and increases the survival and life quality of HIV patients, and greatly reduces the occurrence and severity of opportunistic infections (Santos, 2010; Sunpath *et al.*, 2014; Lindoso *et al.*, 2016). The latter effect has been associated to either a recovery of the immune response or to a direct action of HIV aspartyl peptidase inhibitors (HIV-PIs) on opportunistic pathogens (Santos, 2010; Sunpath *et al.*, 2014; Lindoso *et al.*, 2016). In this sense, HIV-PIs were designed against an aspartyl peptidase from HIV and an orthologous of this enzyme is present at the genome of *Leishmania*. Some data support that this orthologous is the intracellular target for HIV-PIs (White *et al.*, 2011; Perteguer *et al.*, 2013). However, there is no direct evidence on the functional role of the aspartyl peptidases in these parasites, such as knockout or RNA interference approaches. Nevertheless, we and others have demonstrated that HIV-PIs exert potent antiparasitic effects *in vitro* on *Leishmania* promastigotes and intracellular amastigotes, but the exact biochemical target and mechanism of action is still poorly understood (Savoia *et al.*, 2005; Trudel *et al.*, 2008; Santos *et al.*, 2009, 2013*a*; Valdivieso *et al.*, 2010; van Griensven *et al.*, 2013; White *et al.*, 2011; Demarchi *et al.*, 2012 for a comprehensive review see Santos *et al.*, 2013*b*, 2017). Transmission electron microscopy (TEM) revealed that HIV-PIs provoke an increase in lipid inclusions in *Leishmania amazonensis* (Santos *et al.*, 2009). Interestingly, one of the adverse events caused in humans by HIV-PIs treatment is lipodystrophy, which is a condition induced by long-term exposure to PIs that causes excessive fat deposition and accumulation (Hui, 2003). HIV-PIs have been also described modulating proteasome activity (Andre *et al.*, 1998; Schmidtke *et al.*, 1999), apoptosis (Vlahakis *et al.*, 2007; Rizza and Badley, 2008) and lipid metabolism (Hui, 2003; Nolan, 2003; Zha *et al.*, 2013).

Here, considering the possible induction of lipid accumulation by HIV-PIs in *L. amazonensis* (Santos *et al.*, 2009) and the well-established side-effect of these inhibitors on lipid accumulation in treated HIV patients, we decided to investigate whether lopinavir, the most common drug used in clinics, would exert any effect on the lipid metabolism in *L. amazonensis*. Our data indicate the occurrence of a significant accumulation of esterified cholesterol (CHO) in treated parasites in a concentration-dependent manner, culminating in parasite death. These data highlight one of the possible mechanisms of action of this compound.

## Material and methods

### Chemicals and reagents

Lopinavir was synthesized in the Laboratory of Chemical Synthesis, Farmanguinhos, FIOCRUZ, and dissolved in dimethyl sulfoxide (DMSO). Miconazole, heat-inactivated fetal bovine serum (FBS), 7-hydroxy-3H-phenoxazin-3-one-10-oxide sodium salt (Resazurin sodium salt), RPMI-1640 medium, adenine, d-biotin, folic acid, streptomycin, penicillin, hemin, poly-l-lysine, DMSO and lipid standards were purchased from Sigma Aldrich Chemical (St. Louis, MO, USA). ProLong Gold antifade reagent with DAPI (4′,6-diamidino-2-phenylindole) and BODYPI were purchased from Thermo Fisher Scientific (Waltham, MA, USA). Silica plates 60 F254 for thin-layer chromatography (TLC) were purchased from Merck (Frankfurt, DS, Germany). All solvents used were of the purest grade available. All other reagents were analytical grade or superior.

### Parasite culture

Promastigotes of *L. amazonensis* (strain MHOM/BR/77/LTB0016) were cultivated at 26 °C in RPMI medium without phenol red supplemented with 10% FBS, 100 *µ*g mL^−1^ streptomycin, 100 U mL^−1^ penicillin and 5 mg mL^−1^ of hemin, 0.2 mg mL^−1^ of d-biotin, 4 mg mL^−1^ of adenine and 0.5 mg mL^−1^ folic acid.

### Parasites treatment with lopinavir

To evaluate the effect of lopinavir on the lipid metabolism, *L. amazonensis* promastigotes were maintained in flasks at 26 °C for 72 h with the HIV-PI at concentrations ranging from half the IC_50_ (½IC_50_), the IC_50_ and two times the IC_50_ (2 × IC_50_), which correspond to 7.5, 15 and 30 *µ*m, respectively (Santos *et al.*, 2009). Control cells consisted of parasites grown in culture medium with no lopinavir. Parasites treated with the highest DMSO dose used to dissolve the HIV-PI were assessed in parallel for additional control. The tests were performed in culture bottles with an initial inoculum of 1 × 10^6^ parasites mL^−1^. Parasitic growth was evaluated by counting the viable motile parasites in a Neubauer chamber. Parasite viability was assessed by cell motility and trypan blue cell dye exclusion (Strober, 2001).

### BODYPI staining and fluorimetric analysis

*Leishmania amazonensis* promastigotes were treated with lopinavir, as described above. Then, parasites (1 × 10^7^ cells) were washed three times in phosphate-buffered saline (PBS; 150 mm NaCl, 20 mm phosphate buffer, pH 7.2) at 3000 × ***g*** for 10 min at 4 °C and fixed in 4% freshly prepared paraformaldehyde in PBS for 5 min at room temperature (RT). After washing twice in PBS, promastigotes were incubated in 10 *µ*m BODIPY for 30 min at 28 °C, protected from light. Afterwards, parasites were washed three times in PBS (3000 × ***g*** for 10 min at RT) and immediately used in the following experiments. Cellular suspensions were transferred to a black 96-well microplate and BODYPI fluorescence was determined in a Microplate Reader Spectra Max M2 (Molecular Devices): green fluorescence of neutral lipid inclusions was acquired (excitation: 493 nm; emission 503 nm). Alternatively, an aliquot of each cell suspension was collected and adhered to 0.1% poly-l-lysine coated glass coverslips. Samples were mounted in ProLong Gold antifade reagent with DAPI (excitation: 358 nm; emission: 461 nm) and images of neutral lipid inclusions were acquired using appropriated filters in a Zeiss Axio Observer Z.1 epifluorescence microscope coupled to a QImagingRolera EM-C^2^ camera.

### Transmission electron microscopy

Control and lopinavir-treated cells were cultured as described above and promastigotes (2 × 10^8^ cells) were fixed overnight at 4 °C in 2.5% glutaraldehyde in 0.1 m cacodylate buffer, pH 7.2. Thereafter, cells were washed in cacodylate buffer and postfixed for 1 h in 0.1 m cacodylate buffer containing 1% osmium tetroxide, 0.8% potassium ferrocyanide and 5 mm CaCl_2_. Then, cells were washed in the same buffer, dehydrated in acetone and embedded in Epon. Ultrathin sections were mounted on 300-mesh grids, stained with uranyl acetate and lead citrate and observed under a Zeiss 900 TEM (Zeiss, Oberkochen, Germany) (Santos *et al.*, 2009; Sangenito *et al.*, 2014).

### Lipid extraction and TLC

*Leishmania amazonensis* promastigotes were treated with lopinavir (½IC_50_, IC_50_ and 2 × IC_50_) or miconazole (2 and 4 *µ*m, ½IC_50_ and IC_50_, respectively) (Andrade-Neto *et al.*, 2016), miconazole plus lopinavir (2 + 15 *µ*m or 4 + 15 *µ*m) or in culture medium alone for 72 h. After incubation, 1 × 10^8^ parasites from each culture were washed three times with PBS (3000 × ***g*** for 10 min) and their neutral lipids were extracted by the method of Bling and Dyler (Bligh and Dyer, 1959). Briefly, parasites were resuspended in 0.5:2:0.4 parts of chloroform:methanol:water (v/v/v) and homogenized. The suspension was kept under stirring for 1 h at RT and centrifuged (3000 × ***g*** for 20 min) and the supernatant, enriched in lipids, was transferred to a new tube. The pellet was subjected to a second extraction of lipids. The supernatants were added to water:chloroform (1:1), and after 40 s of agitation, the material was centrifuged (3000 × ***g*** for 30 min). The lipid phase was then separated, and the solvent was evaporated using a centrifugal evaporator and resuspended in 50 *µ*L of chloroform. The lipid extracts were spotted onto silica gel TLC plate previously impregnated with silver nitrate (1%) in methanol, to obtain a better separation of the lipids with double bounds, especially ergostane-related sterols from CHO. The plate was run in two steps. The first run, at the half of the plate, was made with hexane:ethylether:acetic acid (60:40:1, v/v/v), and the second was made with hexane:chloroform:acetic acid (80:20:1, v/v/v) (Mangold, 1969). The plates were developed using a charring reagent (CuSO4) followed by heating at 200 °C for 20 min (Bitman and Wood, 1982). After that, the chromatography plates were digitized, and the bands were quantified by densitometry (Andrade-Neto *et al.*, 2011). Cholesterol-ester (CHOE), ergosterol (ERG), CHO, lanosterol, squalene, monoacylglycerol, diacylglycerol (DG) and triacylglycerol (TG) were used as standards.

### Sterol extraction

Total lipids were extracted as described above and dried under a stream of nitrogen. The sterol content was extracted after saponification (Arthington-Skaggs *et al.*, 1999). Briefly, 3 mL of 25% alcoholic potassium hydroxide solution (25 g KOH and 35 mL of sterile distilled water, brought to 100 ml with 100% ethanol) were added to each pellet and vortex-mixed for 1 min. Cell suspensions were transferred to 16 by 100 mm sterile borosilicate glass screw-cap tubes and were incubated in an 85 °C water bath for 1 h. Following incubation, tubes were allowed to cool to RT for 30 min. Sterols were then extracted by addition of a mixture of 1 mL of sterile distilled water and 2 mL of *n*-heptane followed by vigorous vortex mixing for 3 min. The heptane layer was transferred to a clean borosilicate glass screw-cap tube. The sterols were dried with N_2_ and resuspended in 50 *µ*L silylant STFA, TMCS (99:1) + 50 *µ*L pyridine followed by incubation for 1 h at 65 °C.

### Gas chromatography–mass spectrometry analysis of the *L. amazonensis* sterols

*Leishmania amazonensis* sterols were analysed by the use of gas chromatography–mass spectrometry (GC–MS), wherein the lipids were extracted from promastigotes grown in the presence of lopinavir, miconazole or both drugs. The analysis of the sterol fraction by GC–MS was carried out on a Shimadzu GCMS-QP2010 Plus system, using an HP Ultra 2 (5% phenyl – methylpolysiloxane) of Agilent (25 m × 0.20 mm × 0.33 *µ*m). Injector was set at 250 °C. Column temperature was elevated to 50–270 °C with a heating rate of 15 °C min^−1^ and 270–300 °C, with high rate heating of 0.9 °C min^−1^, and held at 300 °C for 6 min. Helium was used as carrier gas with linear velocity of 37.9 cm s^−1^. A volume of 1 *µ*L of sample was injected into the chromatograph. Electroionization (EI-70 eV) and a quadrupole mass analyser were operated in scans from 40 to 600 amu. Interface was set at 280 °C and the ion source at 280 °C. The components were identified by comparing their mass spectra with those of the library NIST05 contained in the computer's mass spectrometer.

### Statistical analysis

All experiments were repeated at least three times in triplicate and the graphics were generated with Graphpad Prism 6 software. Fluorimetric data were analysed statistically using one-way analysis of variance test followed by Tukey post-test. Student's *t*-test was used to evaluate densitometry data. *P* values of 0.05 or less were considered statistically significant. Representative images of these experiments are shown.

## Results

### Promastigote lipid accumulation depends on lopinavir concentration

**Fig. 1.**
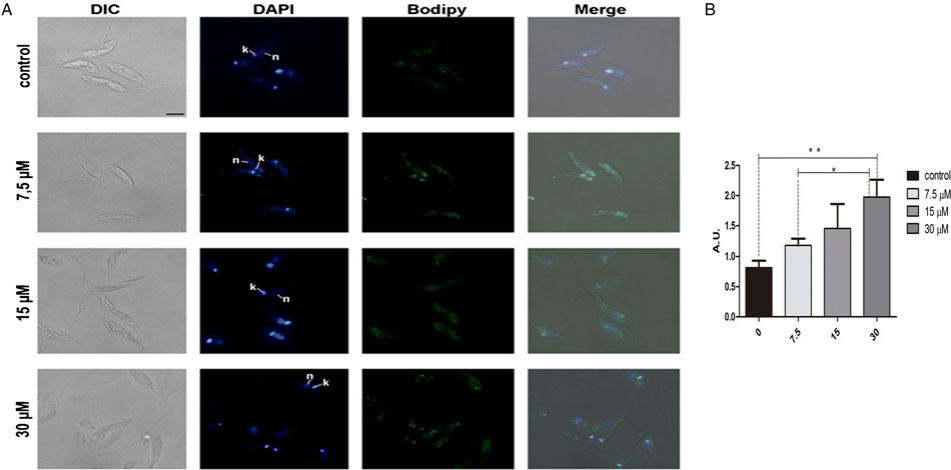
Neutral lipid distribution in *Leishmania amazonensis* promastigotes cultivated in different lopinavir concentrations and incubated with BODIPY. (A) Promastigotes were grown in 7.5, 15 and 30 *µ*m of lopinavir (½IC_50_, IC_50_ and 2 × IC_50_, respectively) for 72 h and incubated with BODIPY and DAPI. Cells were analysed under differential interferential contrast (DIC) and fluorescence in a Zeiss epifluorescence microscope. In the images, the kinetoplast (k) and nucleus (n) are indicated. (B) Fluorimetric analysis using BODIPY revealed that promastigotes incubated with 2 × IC_50_ stored more neutral lipids than those grown in lower concentrations of this inhibitor. Fluorescence intensity was expressed in arbitrary units (AU). The experiments were performed three times in triplicate and data are shown as bar graphs ± standard error of the mean. *P <  0.01, **P < 0.05.

In order to analyse the effect of lopinavir on leishmanial lipid content, *L. amazonensis* promastigotes cells were grown for 72 h in the presence of ½IC_50_, IC_50_ and 2 × IC_50_ concentrations of the compound. Lipid bodies (LB) were distributed throughout the parasite body, as visualized by cell labelling with BODIPY (Fig. 1A). Treated parasites presented a clear increase in green fluorescence intensity in relation to control cells, in a concentration-dependent manner, as revealed by fluorescence fluorimetric measurements. In parasites treated with 2 × IC_50_, there was an enhancement of more than two times of the fluorescence emission, when compared with untreated parasites (Fig. 1B).

**Fig. 2.**
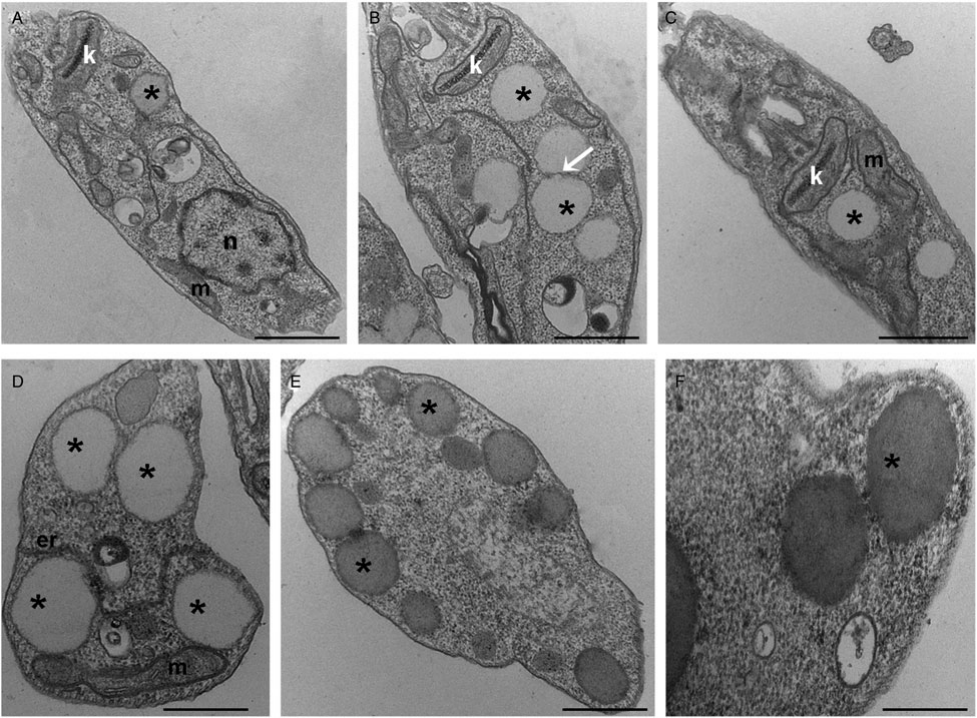
Transmission electron microscopy ultrastructural analysis of *Leishmania amazonensis* promastigotes treated with lopinavir. Untreated parasites (A) or those treated with ½IC_50_ (B), IC_50_ (C and D) and 2 × IC_50_ (E and F) of lopinavir for 72 h are shown. Lipid inclusions (asterisks) are numerous in parasites treated with lopinavir (B–H). Sometimes it is possible to detect fusion between lipid bodies (B – arrow). Such structures were observed in close association with the mitochondrion (C and D) and the endoplasmic reticulum (D). Lipid bodies are commonly observed at the cell periphery (E), close to the plasma membrane and even promoting its protrusion (F). M – mitochondrion, N – nucleus, ER – endoplasmic reticulum. Bars equal to 2 *µ*m (A–C), 1 *µ*m (D and E) and 0.5 *µ*m (F).

To further analyse the lipid inclusions distribution, control and treated parasites were submitted to TEM analysis. After lopinavir treatment, the parasites presented larger lipid inclusions in relation to control cells (Fig. 2A–F). Usually LB were observed very close to each other and often appear to be fusing (Fig. 2B). These structures are seen in association with the mitochondrion (Fig. 2C), as well as with the endoplasmic reticulum (Fig. 2D) and are positioned at the cell periphery (Fig. 2E), sometimes promoting protrusions on the plasma membrane (Fig. 2F).

### Effect of lopinavir on sterol biosynthesis of *L. amazonensis* promastigotes

**Fig. 3.**
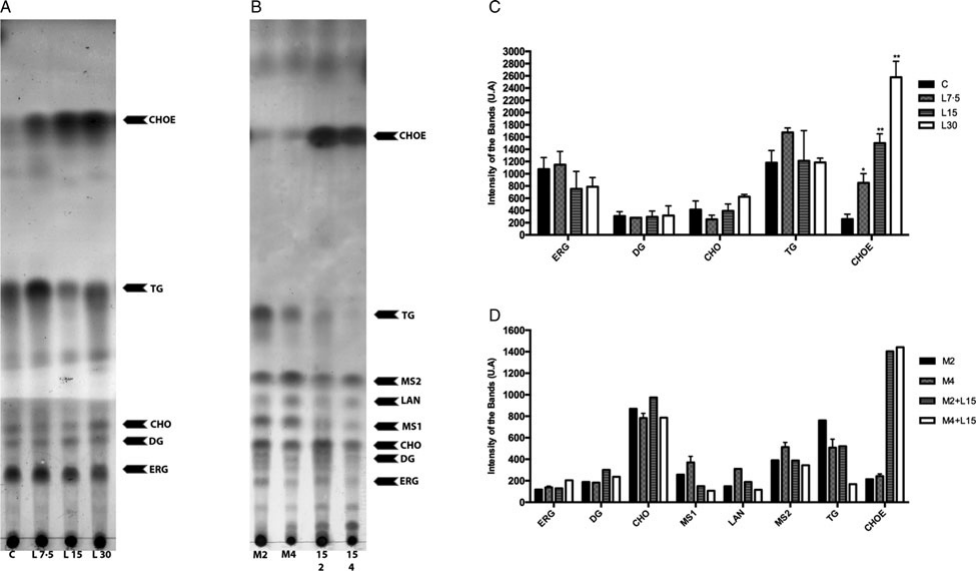
Effect of lopinavir on the lipid composition of *Leishmania amazonensis* promastigotes. (A) *Leishmania amazonensis* promastigotes were incubated in the absence (C – control) or presence of varying concentrations of lopinavir (L7.5, L15 and L30 *µ*m) and (B) miconazole (M2 and M4 *µ*m) alone or in combination (M2 + L15 *µ*m or M4 + L15 *µ*m) for 72 h. Neutral lipids were extracted as described and subjected to thin-layer chromatography (TLC). The TLC image is representative of three independent experiments. Lipid standards used were: ergosterol (ERG), diacylglycerol (DG), cholesterol (CHO), lanosterol (LAN), triacylglycerol (TG) and cholesteryl palmitate (CHOE). The sterol content of parasites treated with lopinavir (C), miconazole alone or miconazole plus lopinavir (D) was estimated by densitometry of the TLC using Image Master Total Lab v1.11 software (AU – arbitrary units). The plotted data are mean standard error of three independent experiments. *P < 0.05, **P < 0.01.

Lopinavir has already been described as effective in inhibiting the growth of *Leishmania* spp. (Santos *et al.*, 2009). Miconazole, an inhibitor of the enzyme sterol C14 demethylase, stops the ERG biosynthesis in *Leishmania*, which causes the accumulation of precursors and an increase in the uptake of exogenous CHO (Andrade-Neto *et al.*, 2011). Thus, miconazole was included in the assay for comparison purposes. The activity of lopinavir on the sterol biosynthesis in promastigotes of *L. amazonensis* was evaluated by TLC after parasite treatment with increasing concentrations of lopinavir, miconazole or the combination of both compounds for 72 h (Fig. 3A and B). Densitometric analysis of the TLC revealed several alterations in the lipid profile after lopinavir treatment, like a marked increase of CHOE (Fig. 3C). However, no significant decrease in ERG content was observed in these parasites and no accumulation of methylated sterols (MS1 and MS2) after lopinavir treatment, as observed in the treatment with miconazole (Fig. 3B and D). The results of GC–MS showed an increase in the CHO content in a dose-dependent manner (Table 1). Since the saponification during the extraction disrupts the ester bonds, the total amount of CHO observed in GC–MS analysis is the sum of free CHO and CHOE. The treatment with miconazole induced an inhibition of the biosynthesis of 5-dehydroepisterol, with accumulation of the methylated sterol, 4,14-dimethylzymosterol (Table 1). Despite the decrease in 5-dehydroepisterol in cells treated with 2 × IC_50_, there was no accumulation of methylated sterols, which corroborates the TLC data.

**Table 1.** GC–MS analysis of sterol composition in *Leishmania amazonensis* treated with lopinavir, miconazole or miconazole plus lopinavir

			Relative amount (%) after treatment							
	MW^a^	R.T.^b^	Control	Lopi 7.5 *µ*m	Lopi 15 *µ*m	Lopi 30 *µ*m	Mico 2 *µ*m	Mico 4 *µ*m	Mico 2 *µ*m + Lopi 15 *µ*m	Mico 4 *µ*m + Lopi 15 *µ*m
Cholesterol	386	25.428	18.79	20.92	22.17	89.57	22.13	26.07	61.78	45.69
Cholesta-5,7-dien-3*β*-ol	384	26.345	0.68	–^c^	0.85	–^c^	–^c^	–^c^	–^c^	–^c^
Ergosta-7,22-dien-3*β*-ol	398	26.768	–^c^	–^c^	–	–^c^	7.89	6.03	–^c^	1.26
Ergosterol	396	27.493	1.20	–^c^	0.75	–^c^	–^c^	–^c^	–^c^	–^c^
Unknown	410	27.722	–^c^	–^c^	–	–^c^	3.66	3.00	–^c^	–^c^
Unknown	396	27.961	1.37	–^c^	0.79	–^c^	–^c^	–^c^	–^c^	–^c^
4,14-Dimethylzymosterol	412	28.033	–^c^	–^c^	–	–^c^	51.78	38.32	24.28	27.29
5-Dehydroepisterol	396	28.627	70.67	70.34	64.51	8.02	4.30	5.25	1.97	4.40
Episterol	398	29.037	5.42	8.74	6.90	2.41	1.36	7.25	4.39	1.18
Lanosterol	426	29.478	–^c^	–^c^	–^c^	–^c^	8.88	12.83	7.58	20.18
Unknown	426	30.488	–^c^	–^c^	–^c^	–^c^	–^c^	0.28	–^c^	–^c^
Stigmasta-5,7,24-trien-3*β*-ol	410	31.558	1.87	–^c^	4.03	–^c^	–^c^	0.97	–^c^	–^c^

a Molecular weight (Da).

b Retention time.

c Non-detectable.

To find out whether the accumulated band (CHOE) was actually CHOE (from an exogenous source) or other endogenous esterified ergostane sterol, parasites were simultaneously treated with lopinavir and miconazole. Interestingly, there was an increase in the CHOE band intensity, even higher than lopinavir alone at the IC_50_ concentration (Fig. 3B). This result confirmed that the sterol accumulated in lopinavir-treated parasites is not an ergosterol-ester, since it persists even when ERG biosynthesis is inhibited by the combined treatment with miconazole and lopinavir. GC–MS and densitometric analysis of TLCs confirmed that this combined treatment induced an increase in CHOE band intensities (Table 1 and Fig. 3D).

## Discussion

Although HIV-PIs are targeted to the retroviral aspartyl peptidase, long-term treatment leads to important side-effects, such as insulin resistance (Hruz, 2008) and lipodystrophy (Hui, 2003; Nolan, 2003). Lipodystrophy is characterized by the abnormal distribution of fat in the human body, increased levels of triglycerides, very low-density lipoprotein and low-density lipoprotein (LDL) CHO (Periard *et al.*, 1999). Considering this important side-effect of HIV-PIs and previous observations from our research group that these inhibitors seem to induce an increase in lipid inclusions in *L. amazonensis* (Santos *et al.*, 2009), we decided to analyse the effect of lopinavir on *L. amazonensis* lipid metabolism.

This study shows for the first time that parasites treated with increasing concentrations of lopinavir exhibited accumulation of lipid inclusions and increased amounts of CHOE in a dose-dependent manner. Interestingly, lopinavir alters the sterol profile in *L. amazonensis* without inhibiting ERG biosynthesis, since this lipid content was not affected by the HIV-PI treatment. Sterol biosynthesis inhibitors have been widely studied in *Leishmania* spp., particularly because this pathway is a promising target for antiprotozoal chemotherapy (Rodrigues *et al.*, 2002; Magaraci *et al.*, 2003; Chawla and Madhubala, 2010; de Macedo-Silva *et al.*, 2015; Andrade-Neto *et al.*, 2016). In this regard, the mode of action of miltefosine on *Leishmania* lipid metabolism has been recently deeply investigated (Armitage *et al.*, 2018). Miltefosine also induces apoptosis-like death and the disruption of metabolite transport. It is possible that these two mechanisms are linked to an interference in the lipid metabolism (Armitage *et al.*, 2018). *Leishmania* treated with miltefosine presented a dramatic decrease in many membrane phospholipids, in addition to amino acid pools, while sphingolipids and sterols increased. This change in the lipid content can alter lipid micro-domain complexes of sterols in the *Leishmania* parasites membranes. In tumour cells, the apoptosis induced by miltefosine is associated to changes in lipid micro-domains. It is also interesting that the analysis of a mutant of *Leishmania major* that lacks the first and rate-limiting enzyme of the biosynthetic pathway of sphingolipids revealed a three times lower susceptibility to miltefosine, while the drug intracellular concentration presented no significant difference in relative concentration in relation to wild-type cells. The increase in the sphingolipid content reported may be related to either a stimulatory effect of miltefosine on the biosynthesis itself or to the inhibition of a catabolic pathway, the latter more probable, since the mutant cells also presented an increase in sphingolipids (Armitage *et al*., 2018).

It has been shown that ERG biosynthesis inhibitors induce alterations in the ultrastructure of some organelles. An important alteration observed after treatment with sterol biosynthesis inhibitors is the presence of several LB displaying variable morphology (de Souza and Rodrigues, 2009). Interestingly, even though the ERG biosynthesis was not targeted by lopinavir, there was a pronounced accumulation of CHOE, which in mammalian cells is the CHO storage form (Maxfield and van Meer, 2010). De Cicco *et al.* (2012) reported the capacity of *L. amazonensis* to synthesize CHOE using LDL particles as the sole source of CHO. Trypanosomatids are able to incorporate CHO from the host or from the culture medium through LDL receptors endocytic pathway (Bastin *et al.*, 1996).

LB are cytoplasmic intracellular inclusions of compartmentalization and storage of lipids. In microorganisms such as fungi, microalgae and bacteria, the accumulation of these LB seems to be induced specifically in response to some stress. The structure and morphology of LB have been described in mammalian cells, especially in adipocytes, and consist of a monolayer of phospholipids, amphipathic glycolipids and/or steroids that surround a hydrophobic core of neutral lipids, mainly TG and esters of sterols (Murphy, 2001; Farese and Walther, 2009; d'Avila *et al.*, 2012; Rabhi *et al.*, 2016; Roingeard and Melo, 2017). Lipid metabolism has been demonstrated as an important pathway modulated by protozoan parasites in host cells. Several studies with *Toxoplamasma gondii*, *Plasmodium* spp. and *L. amazonensis* have documented that these pathogens are able to induce accumulation of lipids, such as TG, DG, CHOE, CHO and/or phospholipids (Coppens *et al.*, 2000; Das *et al.*, 2016; Rabhi *et al.*, 2016; Toledo *et al.*, 2016). Our results suggest that the content of LB observed in the treatment with lopinavir in *L. amazonensis* promastigotes can be composed of CHOE, because the sterol analysis demonstrated an increase of both LB and CHOE in a concentration-dependent manner. In this sense, we suggest that CHO storage (CHOE form) can interfere with the membrane formation.

In conclusion, our findings reinforce that lopinavir can be useful in leishmaniasis treatment and adds some light on one possible mechanism of action, through interference in the sterol metabolism, with an accumulation of LB and an increase in esterified CHO concentration.
